# Human transporter de-oligomerization regulates copper uptake into cells

**DOI:** 10.21203/rs.3.rs-5456520/v1

**Published:** 2024-12-09

**Authors:** Tai-Yen Chen, Meng-Hsuan Wen, Huanhuan Chen, Guangjie Yan, Yuteng Zhang, Wenkai Chen, Martin Dokholyan, Jian Wang, Nikolay Dokholyan

**Affiliations:** University of Houston; University of Houston; University of Houston; University of Houston; University of Houston; University of Houston; Penn State College of Medicine; Penn State University; Penn State College of Medicine

**Keywords:** Oligomerization, CTR1, copper transport, Rab5, endocytosis, single-molecule neighbor density analysis, single-molecule localization microscopy

## Abstract

Copper is an essential element involved in various biochemical processes, such as mitochondrial energy production and antioxidant defense, but improper regulation can lead to cellular toxicity and disease. Copper Transporter 1 (CTR1) plays a key role in copper uptake and maintaining cellular copper homeostasis. Although CTR1 endocytosis was previously thought to reduce copper uptake when levels are high, it was unclear how rapid regulation is achieved. Using single-molecule localization microscopy and single-molecule neighbor density assays, we discovered that excess copper induces monomerization of the wild-type trimeric CTR1 prior to endocytosis, a response blocked in the endocytosis-deficient CTR1 (M150L) mutant. This monomerization rapidly halts copper uptake and prevents copper overload. These findings reveal changes in protein oligomerization as a new paradigm of metal transport regulation, linking CTR1’s structural changes to its endocytosis and copper homeostasis.

## Introduction

Copper (Cu), a metal vital for numerous biochemical processes including electron transfer and the mitigation of oxidative stress, poses a toxicity risk when its cellular levels are not precisely regulated.^1,2^ Copper transporter 1 (CTR1), encoded by the *SLC31A1* gene, stands at the forefront of this regulation as the primary transporter mediating copper uptake from the extracellular environment.^3–5^ CTR1 is an unusual transporter due to its structure and copper-handling mechanism. CTR1 is a homotrimer, which facilitates copper transfer across the plasma membrane without using ATP or secondary ion gradients. Instead, it relies on local Cu concentration gradients and the invariant Met-150 and Met-154 residues for selective copper binding and transport.^6–11^ Although CTR1 looks like a gated channel, its slow permeability rate suggests a transporter-like mechanism, where copper is sequentially bound and then released at the opposite membrane sites through conformational transitions. The rate of copper uptake into the cell is proportional to the CTR1 abundance at the plasma membrane, making the regulation of its surface expression crucial for copper homeostasis.

The regulation of CTR1 abundance at the plasma membrane and thus cellular copper levels is intricately linked to the endocytic trafficking of CTR1. In response to Cu elevation, CTR1 is internalized from the plasma membrane to endosomes.^12–15^ This process, largely mediated by clathrin-mediated endocytosis (CME), is essential for either recycling CTR1 to the plasma membrane as copper levels normalize or targeting it for degradation under persistent copper stress.^13,15^ Whether CME prevents CTR1-mediated Cu transport from endocytic vesicles into the cytoplasm is uncertain. Mutations within the copper-binding motif M^150^xxxM^154^ disrupt Cu uptake and prevent copper-induced CTR1 endocytosis.^16^ By investigating the relationship between copper binding, structural changes in CTR1 and endocytosis, we discovered an unexpected mechanism of regulation of metal ion transport. Specifically, our single-molecule localization microscopy (SMLM) and single-molecule neighbor density (smND) assays revealed the endocytosis associated with de-oligomerization of CTR1, as a rapid mechanism of preventing entry of excess copper.

## Results

### Computational modeling reveals increased stability of CTR1 mutant trimers

To understand how the endocytosis-deficient mutations influence copper uptake, we first employed computational modeling to compare the wild-type CTR1 with the endocytosis-deficient M150 mutant. Using the Eris algorithm,^17^ we simulated changes in CTR1’s thermodynamic stability. In the absence of copper, the leucine substitution at M150 (M150L) reduced the free energy change (ΔΔG) by − 5.11 kcal/mol compared to the wild type. With copper present, where M150L bound one copper at M154 while the wild type bound two coppers at M150 and M154, the ΔΔG of M150L further decreased to − 9.62 kcal/mol. We also examined the alanine mutant M150A, characterized by a shorter side chain and defective CTR1 endocytosis, and found a ΔΔG of − 4.75 kcal/mol without copper. The results suggest that the M150L mutation significantly stabilizes the trimeric form of CTR1, reducing the free energy change compared to the wild type, especially in the presence of copper.

**Figure 1:**
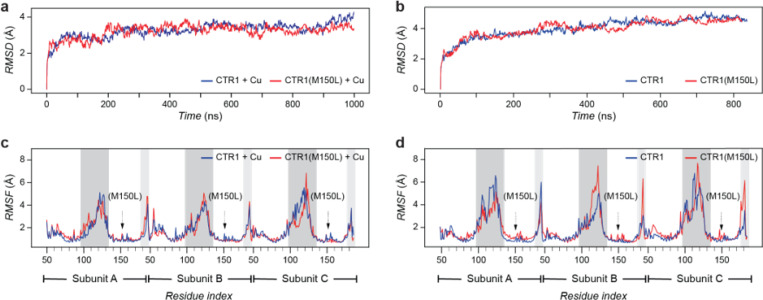
Structural stability and flexibility of CTR1 and CTR1(M150L) analyzed by molecular dynamics simulations. **a, b**, Root mean square deviation (RMSD) of wild-type CTR1 (blue) and CTR1(M150L) (red) in the presence (**a**) and absence (**b**) of copper over 800–1000 ns. CTR1 and CTR1(M150L) show similar overall structural stability. **c**, Root mean square fluctuation (RMSF) of wild-type CTR1 and CTR1(M150L) in the presence of copper shows similar patterns. **d**, RMSF of wild-type CTR1 and CTR1(M150L) in the absence of copper highlights increased flexibility in the M150L mutant, particularly in the intracellular loop (dark gray area) and cytosolic tail regions (light gray area).

We further explored CTR1’s structural dynamics through molecular dynamics simulations in membrane environments. By measuring the root mean square deviation (RMSD) over 800 ns, we found that the overall structure of the CTR1 trimer, for both the M150L mutant and the wild type, remained consistent (Fig. 1a,b). These results indicate that the M150 mutation does not significantly alter the overall structure of the CTR1 trimer, regardless of the presence or absence of copper. The root mean square fluctuation (RMSF) data of both the wild type and the M150L mutant are comparable in the presence of copper (1.639 Å and 1.646 Å for wild-type and M150L CTR1, respectively, Fig. 1c). However, in the absence of copper, the RMSF data showed increased flexibility in M150L (1.842 Å compared to the wild type 1.747 Å), especially within the intracellular loop (residues 98 to 136) and cytosolic tail (residues 176 to 190, Fig. 1d).

Overall, these results show that the main effect of M150 mutation is to stabilize the trimeric structure, which is likely to diminish capacity for the structural transitions required for endocytosis. The increased stability of the trimer in the mutant raises the possibility that the ability of CTR1 to alter its oligomeric state plays a critical role in its regulation of copper uptake.

### The CTR1 fusion with photoconvertible mEos4b retains transport activity, oligomerization, and Cu-induced endocytosis

**Figure 2:**
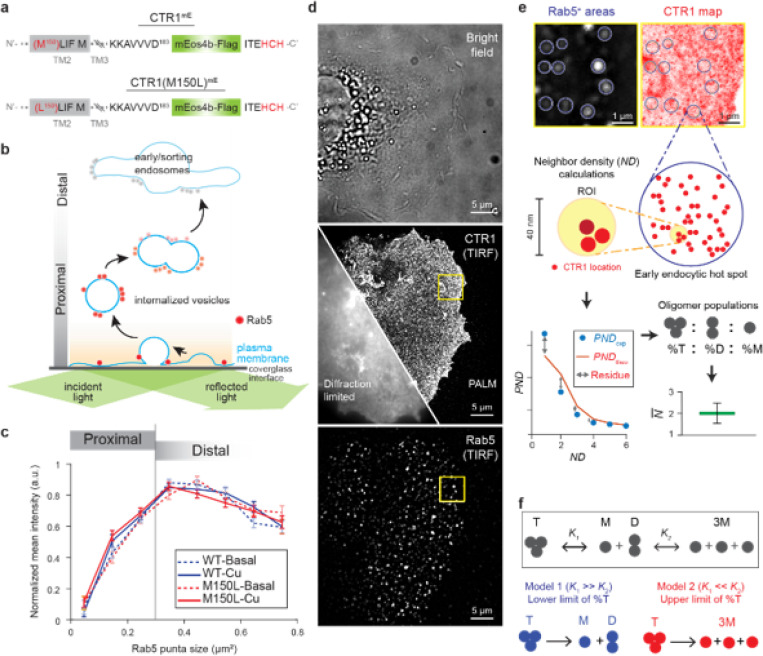
Copper-induced de-trimerization and endocytosis of CTR1. **a**, Schematic representation of wild-type (CTR1^mE^) and mutant (CTR1(M150L)^mE^) constructs. **b**, Diagram illustrating the correlation between Rab5 intensity and endocytic vesicle size using TIRF imaging. Rab5 marks early/sorting endosomes and is present on the internalized vesicles. The intensity of Rab5 is higher in smaller, newly formed endocytic vesicles near the plasma membrane and decreases as vesicles mature and move distally from the membrane. **c**, Normalized mean intensity of Rab5 as a function of Rab5 puncta size in COS7 cells expressing wild-type CTR1^mE^ (WT) and CTR1(M150L)^mE^ (M150L) under basal and Cu-stressed conditions. The graph shows that Rab5 intensity increases with puncta size up to approximately 0.4 μm^2^ and then decreases. There is no significant difference in the Rab5 intensity profile between CTR1^mE^ and CTR1(M150L)^mE^ under either condition, indicating similar early endocytic processes. Graph shows mean ± SEM. Two-way ANOVA. Details of statistical analysis are summarized in Supplementary Table 3. **d**, Representative imaging of CTR1 and Rab5^+^ areas in COS7 cells expressing CTR1^mE^. Bright field image (top), TIRF images of CTR1^mE^ (middle left), and Rab5 (bottom) show diffraction-limited spots. **e**, Simultaneously imaging Rab5^+^ area (left) and CTR1 locations (right) highlights endocytic hot spots, allowing the application of the smND assay to determine CTR1 oligomeric states. CTR1 locations (red dots) within Rab5^+^ areas are grouped to give *ND* if they fall within 40 nm circles. The normalized histogram gives the experimental probability density function of *ND* (*PND*_exp_), which is fitted with theoretical distributions (*PND*_theo_) to obtain CTR1 oligomer populations and $\bar{\text{N}}$. **f**, Models for CTR1 trimer dissociation. Model 1 and Model 2 provide the lower and upper limit of trimer populations, respectively.

The C-terminal HCH motif plays a crucial role in the oligomerization and proper folding of CTR1.^4^ To visualize and investigate the regulation of CTR1 *in situ* without functional perturbation,^18^ we tagged human CTR1 with the photoconvertible fluorescent protein mEos4b (mE), which was inserted into the C-terminus before the HCH motif (Fig. 2a, Supplementary Table 1). The generated CTR1 fusion protein, CTR1^mE^, showed the expected full-length product, confirming the integrity of CTR1^mE^ (Extended Data Fig. 1a). CTR1^mE^ retained copper uptake function and regulation via endocytosis, validated through direct Cu measurement using ICP-MS and surface biotinylation, respectively. Specifically, cells that overexpressed CTR1^mE^ showed significantly higher cellular Cu content (Extended Data Fig. 1b) and reduced surface CTR1^mE^ abundance in the presence of high copper (Extended Data Fig. 1c), indicating effective Cu-induced endocytosis (Extended Data Fig. 2).

To better understand the initiation of CTR1 endocytosis, the small GTPase Rab5, a key regulator of early endocytosis, was used as a marker of endocytic sites at the cell surface. Rab5 coordinates the early steps of CME by interacting with effector proteins to control membrane identity, vesicle formation, and endosome fusion.^19,20^ Activation of Rab5 at the membrane initiates the formation of endocytic vesicles, followed by the vesicle pinched from the plasma membrane. The Rab5 activity on the nascent endocytic vesicles is further reinforced by positive feedback loops involving Rabex-5 and Rabaptin-5. These interactions help stabilize the Rab5 domain on the vesicle membrane, promoting endosome fusion and maturation, which the mature endosomes further move into the cell (Fig. 2b).^21–23^

To examine early endocytic events specifically, we used the total internal reflection of fluorescence (TIRF) imaging to selectively probe proteins near the plasma membrane within a ~ 50 nm depth (Extended Data Fig. 3). Our analysis of Rab5 intensity relative to puncta size showed that smaller puncta (under 0.3 μm^2^, or around 600 nm in diameter) had a positive correlation with Rab5 intensity, indicating vesicle formation. However, as puncta size grew larger than this threshold, Rab5 intensity decreased as vesicles moved out of the TIRF imaging plane, reflecting endosome maturation (Fig. 2c). This bi-phase correlation between Rab5 intensity and puncta size agrees with the Rab5 activation mechanism during the early endosome formation.^22–24^ We therefore focused on Rab5-positive (Rab5^+^) areas smaller than 0.3 μm^2^ to study the roles of CTR1 oligomeric state in the early stages of endocytosis under different conditions.

### The smND assay differentiates proteins in different oligomeric states

Using SMLM under TIRF illumination (Extended Data Fig. 3a), we analyzed CTR1^mE^ within the Rab5^+^ areas (Fig. 2d,e) on the plasma membrane of fixed COS7 cells. This approach captures the early stages of CTR1-involved endocytosis. SMLM was able to resolve individual CTR1 subunits with ~ 10 nm resolution, similar to the experimentally determined CTR1 size (~ 10 nm length and 5 nm width),^10^ and enabled the detection of oligomeric states through our recently developed smND assay (Methods and Supplementary Methods).

The smND assay calculates neighbor density (*ND*) for individual CTR1 subunits, defined as the number of subunits detected within a 40 nm diameter circle. The aggregated *ND* values were visualized as a histogram, representing the experimental probability of neighbor density (*PND*_exp_) distribution and depicting the frequency of encountering various densities of CTR1 subunits (Fig. 2e). We previously developed a theoretical model (*PND*_theo_) to explain how the protein oligomeric state is associated with the *PND* distribution.^25–27^

Using a residue square-based algorithm to match *PND*_exp_ with *PND*_theo_, the smND assay identifies the subpopulations of membrane protein complexes with a typical error of 10% across various test conditions.^25^ This method determines protein oligomeric states without directly resolving entire complexes. Instead, it uses precise localization data to map neighboring protein subunit distributions. By calculating the density of neighboring molecules, smND distinguishes different oligomeric states, such as monomers, dimers, and trimers, based on unique ND distribution patterns. Even when proteins are smaller than the SMLM resolution limit, smND accurately identifies and quantifies their oligomeric states, making it a powerful tool for studying protein behavior in cells.

We noticed that the weighted average number of subunits ($\bar{\text{N}}$), calculated by summing the predicted oligomeric states and their respective percentages (Fig. 2e), can effectively differentiate proteins in different oligomeric states. To validate this, we used the membrane interleukin-2 receptor (Tac-antigen) as a model system. Tac-antigen is known to form disulfide-linked homodimers and exist in a monomer-dimer equilibrium in cells,^28^ making it a suitable system for assessing the sensitivity of our smND assay in detecting different oligomeric states. We generated two constructs by fusing either one or two copies of mEos4b to the Tac-antigen, resulting in Tac^mE^ and Tac^2mE^, respectively. The Tac^mE^ thus represented a mixture of mE monomers and dimers, while the Tac^2mE^ represented a mixture of mE dimers and tetramers. Despite the difference in mEos4b subunit numbers, both constructs have the same Tac monomer-to-dimer ratio, as confirmed by immunoblotting (Extended Data Fig. 4a). The smND population analysis (Extended Data Fig. 4b) further supported these findings, showing larger $\bar{\text{N}}$ values in Tac^2mE^ compared to Tac^mE^, which reflects the increased mEos4b subunits and confirms the accuracy of our approach (Extended Data Fig. 4c). Importantly, since Tac is not involved in copper binding or homeostasis, its oligomeric state is expected to remain unchanged under copper-stressed conditions. Indeed, comparing Tac^mE^ under basal and Cu-stressed conditions revealed no significant difference in $\bar{\text{N}}$, indicating that Cu treatment does not alter the oligomeric state of the mE tag itself (Extended Data Fig. 4d,e).

Compared to dimers, the analysis of trimeric proteins introduces an additional layer of complexity. In such cases, a mixture of monomers and trimers needs to be differentiated from pure dimer (Methods and Supplementary Methods). To achieve accurate identification of trimeric protein subpopulations, we incorporated a dissociation model into the smND assay. This model enables the estimation of the upper and lower boundaries of different oligomeric states by factoring in the dissociation dynamics (Supplementary Methods and Table 2). As depicted in Fig. 2f, the CTR1 dissociation begins with the breakdown of a trimer (T) into a monomer (M) and a dimer (D), marked by an equilibrium constant $K_{1}$. Subsequently, the dimer dissociates into two monomers, regulated by another equilibrium constant $K_{2}$. The $K_{1}$ and $K_{2}$ dictate the relative abundances of different oligomeric forms. Two theoretical models define the dissociation extremes: Model 1, where the ratio $K_{1}/K_{2}$ approaches infinite (i.e., $K_{2}$ approaching zero), posits a single dissociation scenario where trimer dissociation yields a monomer and a dimer (T◇M+D). Conversely, Model 2, where the ratio $K_{2}/K_{1}$ approaches infinite (i.e., $K_{2}$ approaching infinity), suggests that dimeric CTR1 is virtually nonexistent, leading to a direct dissociation of the timer into three monomers (T◇3M). These models offer a framework to estimate the extreme dissociation conditions of trimeric proteins, enhancing our capacity to dissect the intricate oligomeric states of trimeric proteins within the complex environment of the cell (Fig. 2f).

### Cu elevation induces surface CTR1 de-trimerization

**Figure 3:**
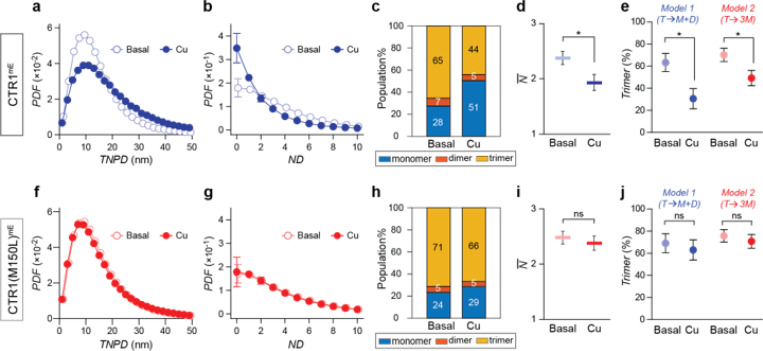
Copper-induced changes in CTR1 oligomeric states assessed by TNPD and smND Assays. a, b, Probability density functions of two nearest pairwise distance (TNPD, a) and neighbor density (ND, b) for CTR1mE under basal (open circles) and Cu-stressed (filled circles) conditions. Copper treatment increases TNPD and reduces ND, indicating larger separation between CTR1 units and de-trimerization of CTR1, respectively. c, d, Extracted oligomer populations (c) and weighted average number of subunits ($\bar{\text{N}}$, d) for CTR1mE show that copper significantly modulated oligomer populations and $\bar{\text{N}}$ in CTR1mE. e, CTR1 trimer percentages extracted from smND with two dissociation scenarios: Model 1 (T→M+D) and Model 2 (T→3M). Model 1 and Model 2 provide the lower and upper limits of trimer populations, respectively. In both models, copper induced significant trimer populations decrease. (f-j) Similar to (a-e) but for CTR1(M150L)mE. No significant change in TNPD distributions (f) nor ND distributions (g). Copper does not affect the extracted oligomer populations (h), $\bar{\text{N}}$ (i), nor trimer population (j) in rab5+ areas, confirming that the M150L mutation locks CTR1 in a trimeric state.Data are summarized from three independent experiments. Graphs (b, d-e, g, and i-j) are shown as mean ± SEM. Student t test. *p < 0.05. See also Supplementary Table 3.

Using CTR1 locations within the Rab5^+^ areas, we first conducted the two nearest pairwise distance (*TNPD*) analysis, leveraging spatial information to infer the oligomeric states of CTR1^mE^ under basal and high copper (100 μM CuCl_2_ for 15 minutes) conditions. We found an increase in *TNPD* for CTR1^mE^ under copper stress, which pointed to increased separation between CTR1^mE^ subunits (Fig. 3a). However, while *TNPD* analysis hints at potential changes, it falls short of distinguishing different CTR1 oligomeric forms. To resolve the CTR1 oligomeric states, we applied smND assay. Fitting the neighbor density (*ND*) distribution (Fig. 3b) showed that the fraction of trimer population decreased from 65 ± 6% to 44 ± 8% (mean ± SEM, Fig. 3c). The corresponding $\bar{\text{N}}$ (Fig. 3d) also decreased from 2.38 ± 0.12 to 1.93 ± 0.13, suggesting that copper induced CTR1 dissociation. To verify de-oligomerization of CTR1, we employed two de-trimerization kinetic models (Fig. 3e). The trimeric population of CTR1^mE^ decreased from 63 ± 8% to 31 ± 9% in Model 1, and from 70 ± 6% to 49 ± 7% in Model 2. Both models suggest that Cu induces a decrease in surface CTR1 oligomerization toward the monomeric form. Since copper elevation is known to trigger CTR1 endocytosis and reduce its surface abundance,^12–15^ this mechanism, together with our findings of copper triggering the dissociation of CTR1 oligomers, suggests that copper-induced oligomeric changes and endocytosis may be coupled.

### CTR1(M150L) variant lacks Cu-induced de-trimerization and endocytosis

Previous studies have shown that the mutation of methionine within the copper-binding motif M^150^xxxM^154^ disrupts copper-induced endocytosis. This motif forms an intramembrane pore essential for copper coordination in the trimeric CTR1 structure.^16^ The M150L mutation, which stabilizes the trimer (ΔΔG=−5.11 and − 9.62 kcal/mol compared to the wild type in the absence and presence of copper, respectively), led us to investigate its impact on CTR1’s oligomer dynamics and its connection to endocytosis.

We created a CTR1^mE^ variant with the M150L mutation (CTR1(M150L)^mE^, Fig. 2a) and characterized its copper transport and endocytosis capacity. Biochemical results of CTR1(M150L)^mE^ showed a deficiency in Cu uptake and endocytosis. ICP-MS data of cells transfected with CTR1(M150L)^mE^ showed no significant changes in intracellular Cu levels compared to mEos4b controls in basal and Cu-stressed conditions (Extended Data Fig. 1b), confirming that the M150L mutation disrupted CTR1-mediated copper uptake. Surface biotinylation studies showed no change in the surface abundance of CTR1(M150L)^mE^ after 30 minutes of copper stimulation for (Extended Data Fig. 1c), consistent with previous findings that the M150 mutation impairs Cu-stimulated endocytosis.

Analyses of *TNPD* and *PND* distributions revealed no significant changes in CTR1(M150L)^mE^’s oligomeric states in response to copper stress (Fig. 3f,g). The smND showed that the extent of Cu-induced de-trimerization for the CTR1(M150L)^mE^ (5–6%) and the change of $\bar{\text{N}}$ ($\Delta \bar{\text{N}}=-0.1$) was sharply reduced relative to the wild-type CTR1^mE^ (21–33% and $\Delta \bar{\text{N}}$ as −0.44, Fig. 3c–e and 3h–j).

These findings indicate that the M150L mutation prevents both Cu-induced de-trimerization and CTR1 endocytosis, supporting the connections between these processes. However, before attributing the observed effects solely to changes in CTR1’s oligomeric states, we examined the broader landscape of cellular endocytosis under copper stress. This is necessary to discern whether copper’s influence exclusively CTR1 or affects more generally the cell’s endocytic machinery.

### Cu stress selectively reduces CTR1 density without affecting global endocytosis

**Figure 4:**
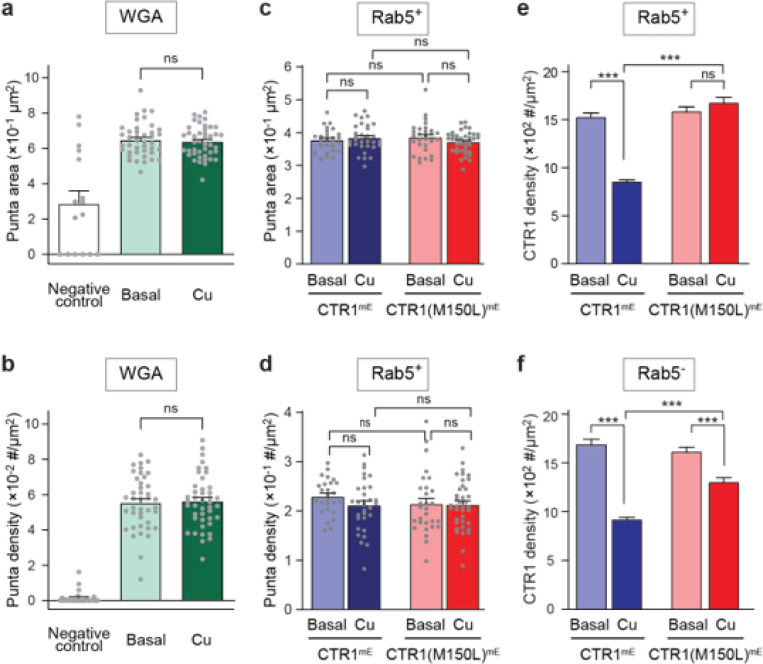
Impact of Cu on global and Rab5-specific endocytosis. **a, b**, WGA-labeled puncta area (**a**) and density (number of puncta/μm^2^) (**b**) in COS7 cells under no endocytosis control (negative control), basal conditions, and Cu-stressed conditions. No significant (ns) difference between basal and Cu-stressed conditions indicates copper does not broadly affect global endocytosis. **c, d**, Rab5^+^ puncta area (**c**) and density (**d**) in COS7 cells expressing CTR1^mE^ and CTR1(M150L)^mE^ under basal and Cu-stressed conditions. No significant difference (ns) between conditions shows copper does not significantly affect Rab5^+^ endosome formation. **e**, **f**, Density of CTR1 within (**e**) and outside (**f**) Rab5^+^ areas in cells expressing CTR1^mE^ and CTR1(M150L)^mE^ under basal and Cu-stressed conditions. Copper significantly reduces CTR1 density in CTR1^mE^ both within and outside Rab5^+^ areas, but this reduction is diminished in the M150L mutant within Rab5^+^ areas and less pronounced outside Rab5^+^ areas. Details of statistical analysis are summarized in Supplementary Table 3. All data are shown as mean ± SEM.

To explore the possibility that copper might influence protein endocytosis globally, we labeled cell surface proteins with wheat germ agglutinin (WGA), a general membrane protein marker, and examined the WGA uptake after copper treatments. The same copper treatment (100 μM CuCl_2_ for 15 minutes) that triggers endocytosis of CTR1 did not significantly increase in global endocytosis, as evidenced by unchanged sizes and numbers of WGA-positive puncta (Fig. 4a,b).

We then focused on Rab5 GTPase, a marker for early endosomes^23,29^ and examined the steady-state distribution of Rab5 on the plasma membrane in COS7 cells expressing either the wild-type CTR1 or the M150L mutant after copper treatment. No significant changes were observed in the Rab5^+^ puncta size or number, suggesting that neither copper nor CTR1 expression altered Rab5-associated endocytic processes (Fig. 4c,d).

Under copper stress, the wild-type CTR1 density decreased by 45% in Rab5^+^ areas, suggesting effective internalization through Rab5-associated pathways (Fig. 4e, left). In contrast, while the M150L mutant still colocalized with Rab5^+^ areas, the M150L mutant showed no reduction in density (Fig. 4e, right), indicating that although the mutant can associate with Rab5-labeled endocytic hot spots, it is unable to be internalized.

Outside of Rab5 area (Rab5^−^) areas, the wild-type CTR1 also showed a 45% reduction in density (Fig. 4f, left). This decrease could result from the Rab5^+^ pathway reducing overall surface CTR1 levels, affecting both Rab5^+^ and Rab5^−^ regions. Alternatively, this reduction may also suggest direct internalization through a Rab5-independent pathway or the combination of both Rab5^+^ and Rab5^−^ pathways. However, the fact that the M150L mutant exhibited a smaller, 19% decrease in Rab5^−^ areas (Fig. 4f, right) suggests that a Rab5-independent pathway also contributes to the reduction in CTR1 density. This highlights the involvement of both Rab5-dependent and Rab5-independent mechanisms in CTR1 internalization, though the latter appears to be less efficient.

## Discussion

### Missing players in copper driven CTR1 de-trimerization and endocytosis

Our smND results (Fig. 3c–e) demonstrate that CTR1 exists in a dynamic equilibrium of different oligomeric states and shifts toward the monomeric state in response to elevated copper levels, which trigger endocytosis. While molecular dynamics simulations suggested that both the M150 mutation and copper binding at specific sites (M150 and M154) stabilize CTR1 trimers, experimental observations revealed a contrasting phenomenon: copper induces de-trimerization of wild-type CTR1 at early endocytic areas, a response blocked in the M150L mutant.

Building on this observation, a plausible model is that intracellular copper, entering through CTR1, is detected by a sensor protein that then recruits endocytic components to CTR1’s intracellular loop. The YxxM motif at the intracellular loop of CTR1 has been proposed as the docking site for the endocytic complex.^30^ The binding of this endocytic machinery to the YxxM motif may physically pull the trimer apart, triggering the shift to a monomeric state. This model aligns with our data and explains why copper binding to methionine residues in CTR1 stabilizes the trimer. Since copper is coordinated by these methionine sites, it reinforces the trimeric structure, preventing premature dissociation. Moreover, this model reconciles the rapid de-oligomerization we observe with the known kinetics of copper transport and endocytosis. Copper transport through CTR1 occurs faster than the recruitment of endocytic components, meaning the trimer remains stable during copper uptake. Once copper has entered the cell, endocytic machinery is recruited, causing the dissociation of the trimer. In the absence of copper transport, this recruitment process does not occur, and the CTR1 trimer remains intact, as seen with the M150L mutant, where the mutation disrupts copper sensing and subsequent endocytosis.

### Benefits of CTR1 de-trimerization in copper regulation

It is well known that CTR1 internalization is mainly through clathrin-coated vesicles which emerge at the plasma membrane as clathrin-coated pits (CCPs) and later migrate into cytoplasm after scission. Studies using TIRF microscopy to track CCPs have revealed that a significant proportion of clathrin-coated structures on the plasma membrane were in the “before scission” stage due to their longer lifetimes. This stage includes the recruitment of clathrin and adaptor proteins, invagination of the plasma membrane, and assembly of the coated pit. After scission from the plasma membrane, these vesicles stay within 200 nm of the membrane for just a few seconds, and then move deeper into the cytoplasm beyond the TIRF detection field.^31–34^ Given the brief surface duration of nascent clathrin-coated vesicles at the surface, we estimate that most CTR1 (> 90%) detected in our system under steady-state conditions were the populations remaining on the plasma membrane before being internalized. Therefore, our observation of CTR1 de-trimerization is likely captured events in this longer-lasting “pre-scission” phase, suggesting that de-trimerization is a faster process than endocytosis.

From a broader perspective, the ability of CTR1 to dissociate into monomers under high copper conditions serves as a rapid regulatory mechanism to prevent copper overload by reducing the number of active transport sites. However, if these monomers were to persist on the plasma membrane, they could potentially reassemble into trimers, which would counteract the regulatory effort. This is where endocytosis plays a crucial role. Endocytosis mitigates this risk by removing the monomers from the membrane, ensuring they don’t reassemble and further limiting copper uptake.

**Figure 5:**
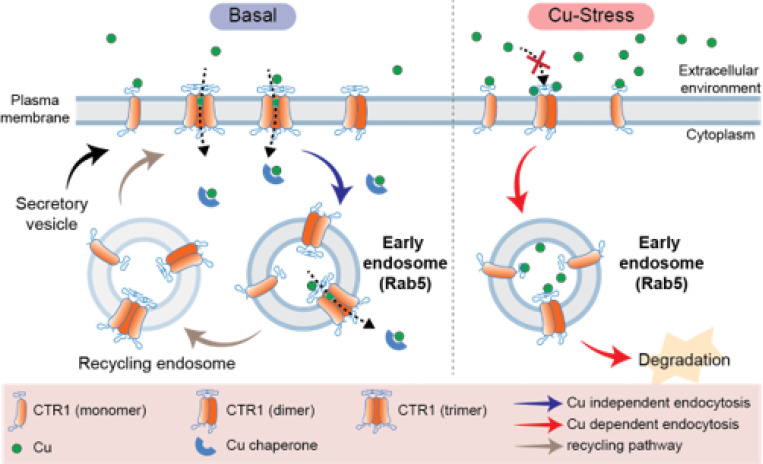
Proposed model of CTR1 oligomeric state modulation and endocytosis under basal and copper-stress conditions. (Left) Under basal conditions, CTR1 exists in a dynamic equilibrium among various oligomeric states, predominantly as trimers on the plasma membrane. These trimers engage in copper-independent, constitutive endocytosis while retaining their channel properties to facilitate copper transport to intracellular chaperones. Post-internalization, CTR1 can recycle back to the plasma membrane or be rerouted to the ER-Golgi network and released via secretory pathways. (Right) In environments with excessive copper, the initial response involves halting copper uptake at the plasma membrane through CTR1 de-trimerization. This is followed by copper-induced endocytosis, removing non-trimeric CTR1 from the cell surface as a secondary defense to contain copper within membrane compartments. It is important to note that non-trimeric CTR1 also prevents copper transport from vesicles into the cytosol. Subsequently, CTR1 is delivered to lysosomes for degradation, effectively excluding copper from the cell.

Additionally, copper can be transported into the cytoplasm across the lipid-bilayer either at the plasma membrane or at the internalized vesicles when functional transporters are still present on the membrane (Fig. 5).^35,36^ Therefore, de-trimerization of CTR1 within endocytic vesicles could prevent copper within the vesicle from entering the cell, providing another layer of copper regulation. These observations highlight that CTR1 not only transports copper but actively adjusts its structural configuration to manage copper levels effectively, particularly at early endocytic areas. The de-trimerization links oligomeric changes directly to endocytic processes essential for copper homeostasis,^37^ underscoring the critical role of structural dynamics in CTR1 function.

### Limitations of the study

This study offers key insights into CTR1’s structural dynamics using SMLM and smND assays, which allow us to resolve CTR1 oligomeric states with high spatial accuracy *in situ*, linking these changes to copper regulation and endocytosis. The ND distribution indicates that most CTR1 subunits have fewer than five neighbors, effectively ruling out the possibility of clustering that could distort the results. Our controls using Tac^mE^ and Tac^2mE^ also confirm that the mEos4b tags do not interfere with oligomerization, ensuring that the observed CTR1 states are genuine and not artifacts of tag interactions.

Despite these strengths, one notable limitation of our study is that all experiments were conducted in fixed samples, which restricts us to quantifying only the steady-state status of CTR1 complexes and cannot capture real-time dissociation kinetics. This restricts our ability to observe the dynamic transitions of CTR1 during copper uptake. Addressing these limitations in future studies will be crucial to fully understand CTR1’s role in copper regulation.

## Methods

### RESOURCE AVAILABILITY

#### Lead contact

Further information and requests for reagents should be addressed to the lead contact, Dr. Tai-Yen Chen (tchen37@central.uh.edu)

#### Materials availability

All reagents described in this manuscript are available upon request from the lead contact with the appropriate Materials Transfer Agreement.

#### Data and code availability

The original code and any additional information required to reanalyze the data reported in this paper are available from the lead contact upon request.

### EXPERIMENTAL MODEL AND SUBJECT DETAILS

#### Human cell lines

HEK293 and COS7 cells were purchased from ATCC for all the cellular experiments performed in the study. The cell lines were examined frequently under the microscope for proper morphology, but they were not authenticated. They were maintained in Dulbecco’s Modified Eagle’s Medium (DMEM) (Gibco 11965126) supplemented with 10% fetal bovine serum (SAFC #12306C), 1× GlutaMax (Gibco 35050061), and 1× sodium pyruvate (Gibco 11360070), at 37°C in a 5% CO2 ambient humidified incubator.

### METHOD DETAILS

#### General reagents

Most of the general reagents were obtained from Sigma Aldrich and Thermo Fisher Scientific unless otherwise mentioned.

#### Plasmid constructions and sample preparation

##### DNA plasmid construction

The internal tagged CTR1^mE^ construct was generated by directly engineering the source hCTR1 plasmid (RC201980) which originally harbored human CTR1 fused with a Myc-DDK-tag at the C-terminus. The coding region of CTR1 (amino acid 185–190) and the following Myc-DDK-tag in the source plasmid was first removed by *Eco*RV digestion and replaced with the polymerase chain reaction (PCR)-amplified mEos4b-Flag fragment (Oligo #1 and #2) by using the NEBuilder HiFi DNA Assembly Cloning Kit (New England Biolabs E5520S). The resulting plasmid was linearized again by *Pme*I digestion, and the coding region of hCTR1 amino acids 185–190 with a stop codon was then restored at the C-terminus of the mEos4b-Flag through single-strand DNA assembling (Oligo #3). This modification resulted in a final construct with mEos4b-Flag internal insertion and kept ^188^HCH domain at the fusion protein’s C-terminus.

The Cu-transfer deficient mutant construct, CTR1(M150L)^mE^, was further generated by site-directed mutagenesis (Oligo #4 and #5) using Q5 site-directed mutagenesis kit (New England Biolab E0554S) following the manufacturer’s instructions.

Plasmids expressing membrane-anchored single or tandem mEos4b were constructed to validate the smND assay. First, the Tac-mEos4b (Tac^mE^) construct was generated by inserting a PCR-amplified mEos4b-Flag fragment into a pcDNA3.1/Zeo(−) plasmid which harbored the interleukin-2 receptor α (Tac) extracellular and transmembrane domains (amino acids 1–262, a gift from Dr. C.-Y. Tai^38,39^) between EcoRI and BamHI. Then another mEos4b fragment was PCR amplified and cloned into the Tac^mE^ plasmid between NotI and EcoRI to generate the Tac-mEos4b-mEos4b (Tac^2mE^) construct. All plasmids were confirmed by Sanger sequencing prior to use.

##### Cell preparations and transfection

For imaging experiments, 1.5 × 10^5^ COS7 cells were transfected with 1 μg plasmid DNA, either CTR1^mE^ or CTR1(M150L)^mE^ using Lipofectamine 2000 (Invitrogen 11668019) for 1 day. The next day, the cells in a 35-mm dish were detached using 300 μl TrypLE Express (Gibco 12604013) and then re-plated onto a custom-made, poly-L-lysine (Sigma P2636) coated glass-bottom imaging chamber made by no. 1.5H cover glass at a density of 10,000 cells/cm^2^ and grown in complete DMEM medium without phenol red (Gibco 31053028) for 2 days. Before imaging, cells were treated with or without 100 μM CuCl_2_ (Sigma C3279) in a complete growth medium for 15 min to induce CTR1 internalization. Cells were then fixed in 4% paraformaldehyde (Electron Microscopy Sciences 15710) in PBS at room temperature for 15 min.

##### Immunofluorescence staining and immunoblotting

For immunofluorescence staining, COS7 cells transfected with indicated plasmids were grown on a poly-L-lysin coated glass-bottom imaging chamber with 10,000 cells/cm^2^. Following specific copper treatments, cells were washed 3 times with ice-cold phosphate buffer saline (PBS) and fixed in 4% paraformaldehyde/PBS at room temperature for 20 min. Cells were permeabilized with 0.1% Triton X-100 prepared in the blocking buffer (2% bovine serum albumin (Sigma A7906), 4% normal goat serum (Gibco 16210064) in PBS) at room temperature for 20 min, and then further incubated in the blocking buffer for 30 minutes, followed by incubated with anti-Rab5 antibody (C8B1, Cell Signaling Technology 3547; 1:200 dilution) overnight at 4°C. After 3 times of wash with PBS, cells were probed with Alexa Fluor^™^ 633-conjugated secondary antibodies (Thermo Fisher Scientific A-21071; 1:500 dilution) for an additional 2 h at room temperature. After three final washes with PBS, the cells were ready for imaging.

For immunoblotting, cells were harvested in the lysis buffer (25 mM Tris pH 7.4, 150 mM NaCl, 0.1% SDS, 1% Triton-X-100 and 1x Halt Protease inhibitors cocktail (Thermo Scientific)) and incubated on ice for 60 min for collecting total proteins. Alternatively, the Mem-PER^™^ Plus Membrane Protein Extraction Kit (Thermo Scientific) was employed for collecting subcellular fractionated proteins. Cell lysates were further centrifugated at 16,000 ×g for 15 min to remove insoluble substances. The protein concentrations were determined by BCA protein assay. Equal amounts of protein were prepared in Laemmli SDS sample buffer containing 2.5% β-mercaptoethanol, heated at 70°C for 5 min, and resolved in SDS-PAGE followed by transferred onto a PVDF membrane for immunoblotting. Anti-Flag (Rockland, 1:2500 dilution) or anti-SLC31A1/CTR1 (Abcam, 1:1000 dilution) were used as primary antibodies and horseradish peroxidase (HRP)-conjugated anti-mouse or -rabbit IgG (Jackson ImmunoResearch Lab, 1:10000 dilution) was used as the secondary antibody. The blots were developed in Immobilon^™^ Western Chemiluminescent HRP Substrate (Millipore) and visualized by using ChemiDoc Imaging System^™^ (BioRad). The same blots were stripped and re-probed with anti-β-Actin (Sigma-Aldrich A5441, 1:5000 dilution) as a loading control. The band densitometry were quantified by using ImageLab Software^™^ (BioRad) or ImageJ (NIH).

#### Functionality assays

##### Surface biotinylation

To measure the abundance of the CTR1 protein on the cell surface under various stress conditions, HEK293 or COS7 cells were transfected with tagged constructs (i.e., CTR1^mE^ or CTR1(M150L)^mE^) using Lipofectamine 2000 (Invitrogen 11668019) following the manufacturer’s instructions. After 48 hours of transfection, cells were treated with either CuCl_2_ or bathocuproinedisulfonic acid (BCS) to a final concentration of 50 μM or 100 μM, respectively, in the culture medium for 30 min.

After treatment, surface proteins were labeled by 0.5 μg/mL EZ-Link^™^ sulfo-NHS-SS-biotin (Thermo Scientific 21331) on ice for 30 min. Cells were rinsed with an excess amount of 200 mM glycine to quench the excess reactive amine group on ice for an additional 15 min. The cells were then harvested in the lysis buffer (0.1% SDS, 1% Triton-X-100, and 1X Halt Protease inhibitor cocktail in PBS), followed by incubation at 4°C with rotation for 60 min. For each 35-mm dish, 250–300 μL of lysis buffer was used, while for each 60-mm dish, 500 μL of lysis buffer was applied. The insoluble material was removed by centrifugation at 16,000 ×g for 15 min. Protein concentrations were determined by BCA protein assay.

Equal amounts of total proteins from each sample were incubated with 50 μl Pierce^™^ NeutrAvidin^™^ agarose beads (Thermo Scientific 29200) at 4°C overnight with rotation to bind the biotinylated proteins. The beads were washed with 1 mL lysis buffer three times to remove non-specific binding. The bound proteins were dissociated in 30 μl of 2X Laemmli SDS-sample buffer and resolved by immunoblotting, using an anti-Flag polyclonal antibody (Rockland 600–401-383) to detect the tagged CTR1 proteins. The same blots were re-probed with an anti-Na/K ATPase α1 (C464.6) monoclonal antibody (Santa Cruz sc-21712) as a loading control.

##### WGA surface labeling

To identify newly internalized vesicles that originated from the plasma membrane, we labeled cells with Alexa Fluor^™^ 555 or Alexa Fluor^™^ 647-conjugated WGA (Invitrogen W32466 or W32466) following the manufacturer’s instructions before inducing endocytosis. In preparation, cells expressing CTR1^mE^ or CTR1(M150L)^mE^ were grown in serum-free and phenol red-free DMEM medium (Gibco 31053028) with 50 μg/mL cycloheximide (CHX, Sigma C1988) for 1 hr at 37°C. This treatment halted new protein synthesis and was followed by chilling on ice to suspend ongoing endocytosis. Cells were rinsed twice with ice-cold HBSS (Gibco 14175095) and incubated with WGA at a concentration of 2.5 μg/mL in HBSS on ice for 10 min. After removing excess staining solution with three rinses in ice-cold HBSS, the cells were incubated in complete growth medium containing serum and either just CHX (for basal conditions) or CHX plus Cu (for Cu-stressed conditions) to trigger endocytosis at 37°C for 15 min. Cells were then rinsed twice with ice-cold PBS and fixed with 3% paraformaldehyde (Electron Microscopy Sciences 15710) and 0.1% glutaraldehyde (Electron Microscopy Sciences 16365) in PBS. Approximately 1–1.5 mL of fixation buffer was applied to the 35-mm glass bottom dish, ensuring it completely covered the surface. After fixation, the cells were washed again with PBS before imaging using a wild-field epi-fluorescence microscope (Olympus CKX53) under 40× Objective (Olympus LUCPLFLN40XRC) for quantifying global endocytosis. The success of WGA-labeling and detecting internalization was demonstrated by the representative single section images taken by using confocal microscopy (Olympus FV3000).

##### Inductively Coupled Plasma Mass Spectrometry (ICP-MS)

HEK293 cells expressing CTR1^mE^ or CTR1(M150L)^mE^ were used to quantify the intracellular Cu levels. HEK293 cells were exposed to 50 μM CuCl_2_ for either 30 or 60 min. After treatment, cells were scraped into PBS and collected by centrifugation at 1000 ×g for 5 min. The cell pellet was digested in 50 μL of ultrapure 70% HNO_3_, heated at 70°C for 30 min and then at 95°C for an additional 2 hrs. Samples were left to cool down naturally to room temperature overnight to achieve complete mineralization.

Mineralized samples were further diluted in ultrapure water to ensure the final HNO_3_ concentrations were below 2%. These samples were then analyzed using a triple quadrupole inductively coupled plasma–mass spectrometry (Agilent 8800 ICP-QQQMS). The analysis focused on isotopes ^63^Cu and ^65^Cu in helium MS/MS mode, and ^32^S and ^34^S in oxygen MS/MS mode, to ensure precise detection. Copper concentrations were determined against standard curves and the values obtained were normalized to the total sulfur content within the same samples, as assessed by ICP-MS, to ensure accuracy in quantification.

#### Structural conformation modeling and thermodynamic simulation analysis

##### Modeling and optimization of human CTR1 trimer structure

The copper transporter is a highly conserved protein across different species,^7,10,11^ with the protein sequences of human CTR1 and *Salmo salar* (Atlantic salmon) Ctr1 being 77% identical. This high degree of conservation makes the X-ray structure of *Salmo salar* Ctr1 (sCtr1) an excellent template for modeling the human protein. The X-ray structure of sCtr1 provided an excellent template for modeling human protein. To construct the trimeric structure of human CTR1 and its relevant mutants, we used the monomeric structure of sCtr1 (PDB ID: 6M97 for the copper-free form and 6M98 for the copper-bound form, with Cu^+^ present at the locations corresponding to human CTR1 M150 and M154) as templates for the SWISS-Model.^7,40^ Using PyMol,^42,46–48^ we aligned three copies of the predicted monomeric human CTR1 structure to the trimeric complex of sCtr1. This alignment resulted in a modeled trimeric structure of human CTR1, leveraging the structural information from the highly conserved sCtr1 to provide a reliable representation of the human protein.

Note that the published sCtr1 has an N-terminal (residues 1–40) truncation and a thermostabilized apocytochrome BRIL replacement at the intracellular loop (residues 94–120) of the original sCtr1 polypeptide.^7^ To accurately represent the human CTR1 structure, we removed the extra polypeptide of BRIL and added back the missing residues in the crystal structure using Modeller.^41^

To ensure the structural viability of the human CTR1 model, an extensive structural integrity analysis was conducted using the Gaia server,^48^ which revealed multiple steric clashes among atoms. To address these issues and ensure the model’s viability for further studies, Chiron^42^ was employed to efficiently address and resolve these clashes, optimizing the modeled CTR1 for further studies.

##### Evaluating CTR1 stability through computational mutagenesis

In the study of CTR1’s thermodynamic stability, computational mutagenesis was employed using the software Eris,^17^ a sophisticated tool designed for virtual mutagenesis and thermodynamic analysis. We used Eris to substitute the amino acid methionine at position 150 with leucine (M150L) and alanine (M150A) to assess their impact on the protein’s stability. The method hinges on the calculation of the change in free energy (ΔΔG) relative to the wild-type CTR1, providing a quantitative measure of the mutations’ effects.

Eris executes this analysis by initially repacking the side chains of the mutated sites and then employing discrete molecular dynamics (DMD) simulations to optimize the protein’s entire structure.^49–52^ Eris calculates the free energy of the entire structure before and after the simulation. The discrepancy in free energy values serves as an indicator of how each mutation influences the overall stability of CTR1.

##### Molecular Dynamics Simulations

To simulate CTR1 in a cellular context for analyzing CTR1 protein dynamics in membrane environments, we embedded CTR1 into a membrane and added counterions using Charmm-GUI.^43^ The membrane boundaries are between residues A66 and S97, Q125 and M154, and G158 and V181 on each chain of the trimer. The constructed membrane comprised of 80% POPC (1-palmitoyl-2-oleoyl-sn-glycero-3-phosphocholine) and 20% cholesterol, reflecting a biologically relevant composition.

Molecular dynamics simulations were performed using the PMEMD.CUDA program in Amber 18.^44^ The simulations utilized a 2-fs time step, maintained the conditions of constant pressure (1 bar), and achieved the temperature (300 K) by using the isothermal-isobaric (NPT) ensemble. To ensure charge neutrality in the system, sodium ions were introduced, along with additional Na^+^ and Cl^−^ ions, to achieve a physiological ion concentration of approximately 0.15 M.

The initial simulation phase spanned 50,000 steps, equivalent to 100 ns, providing preliminary data on root-mean-square fluctuation (RMSF) and unadjusted root-mean-square deviation (RMSD) metrics. Subsequently, the first 50 ns of RMSD data were discarded to allow for system equilibration. The simulation was then extended by an additional 800 ns, enabling a more precise determination of RMSD and RMSF values. These measurements were computed using the Cpptraj tool within the Amber 18 framework, facilitating a comprehensive analysis of protein dynamics and stability within the membrane environment.

#### Single molecule localization microscopy (SMLM) and image analysis

##### Microscope setup

All single-molecule fluorescence experiments were conducted on an inverted fluorescence microscope (Olympus IX83) with the excitation and emission light paths detailed in Extended Data Fig. 3a, and Supplementary Table 1. In the excitation path, lasers with wavelengths of 405 nm, 488 nm, 552 nm, and 640 nm (Coherent, Inc.) were coaxially aligned using a series of long-pass dichroic mirrors (Chroma Tech. Co., ZT458-UF1, ZT488-UF2, and ZT532rdc-UF2). The laser power density for each wavelength was finely tuned using a combination of a half waveplate and a polarizer pair, optimizing conditions for the activation and detection of single molecules. These lasers were directed into the microscope via the backport and focused through a high numerical aperture objective (Olympus, UAPON 100X, N.A. = 1.49) to excite the fluorescent dyes or proteins under study. The typical laser spot size at the sample focal plane is ~ 60 μm in diameter. The power density of 405, 488, 552, and 640 nm lasers is 0.0708 W/cm^2^, 513 W/cm^2^, 184 W/cm^2^, and 251 W/cm^2^, respectively. In the emission path, the fluorescent signals from the excited fluorescent dyes or proteins were then collected by the objective and relayed to a highly sensitive CMOS camera for detection (Teledyne Photometric Inc.).

##### Quantifying TIRF illumination depth

To accurately quantify the evanescent field strength of our TIRF microscopy, we employed the raisin-cake scanning method (Extended Data Fig. 3b), which involves plotting field strength against z-depth to reveal the penetration depth of the evanescent field.^53^

We used 100 nm gold nanospheres as location markers, utilizing their scattering intensities to indicate evanescent field strength. The calibration slide was prepared by mixing 200 μL of a sonicated gold nanosphere solution with 1% agarose gel (0.005 g agarose in 500 μL ultrapure water). This mixture was then heated for 30 seconds in a microwave with a Petri dish water bath and allowed to cool to room temperature, ensuring even distribution and stability of the markers for imaging.

Imaging began with bright-field microscopy to identify the rough lateral locations of gold nanospheres on the glass coverslip surface. The z-location of each gold marker was then determined by re-imaging the sample under fluorescent mode with a 488 nm laser in epi-illumination, scanning from the bottom to the top of a 3 μm range in 10 nm steps. The scattering intensity of each gold nanosphere at each z-location was recorded. Summing the z-image stack from the epi movie revealed a clear point spread function (PSF) for each gold nanosphere. We calculated the center in the x and y dimensions and analyzed the mean intensity within a 3×3 pixel area along the z-stack to construct an intensity versus z trajectory. This trajectory’s peak represents the tightest focus of each gold nanosphere. To obtain the precise z location, each trajectory was fitted with a Gaussian function (Extended Data Fig. 3c). This single nanosphere-based analysis was repeated for approximately 84 gold nanospheres within the 400 nm-wide field of view.

To correct the slight tilt in our system (Extended Data Fig. 3d), we fit the z locations with the equation “$z_{0}=\text{a}x+\text{b}y+\text{c}$” and subtracting $z_{0}$ from all gold nanospheres. For assessing the evanescent field strength, the calibration slide was re-imaged under TIRF mode near the $z_{0}$ plane. We conducted a 500 nm z-scan under TIRF configuration to identify the brightest gold nanospheres, which helped eliminate the influence of inhomogeneous objective focus volume and the tilted coverslip. After identifying the gold nanospheres with the brightest intensity at a given z from the z stack, we obtained the mean intensity from a 3×3 pixel area.

To estimate the noise level at the selected z plane, we created a histogram of the entire image’s intensity and selected the bottom 14% of pixel intensity to calculate the standard deviation of the intensity distribution. We then computed the signal-to-noise ratio (SNR) and used it as an indicator of field strength. Combining the z information from epi-illumination and SNR from TIRF illumination, we generated a field strength versus z-depth plot (Extended Data Fig. 3e). The SNR data were binned in 100 nm intervals and fitted with a single exponential decay convoluted with a Gaussian function. The final penetration depth was determined to be 50 nm, providing a precise measure of the evanescent field strength.

##### Image and localize surface CTR1 and Rab5 positive areas

To specifically probe surface CTR1 and correlate their oligomeric state distribution to membrane trafficking hot spots (Rab5^+^ areas), we simultaneously determined the CTR1 and Rab5^+^ areas localizations using two-color fluorescence microscopy with total internal reflection (TIR) excitation. The TIR angle was controlled by a translational stage (Thorlabs Inc. LST300) on the back of the microscope. This setup enabled selective excitation of surface CTR1 and Rab5 by creating evanescent fields approximately 50 nm deep, ensuring targeted illumination of these proteins.

Surface CTR1 locations were identified using photoactivated localization microscopy (PALM), achieving a typical lateral resolution of ~20 nm. The CTR1^mE^ was activated by a 405 nm laser (power ranging from 0.0708 W/cm^2^ to 7.08 W/cm^2^, gradually increased as needed) and simultaneously excited with a 552 nm laser at 184 W/cm^2^. The emitted red emission from mE was collected by the objective, filtered by a bandpass filter (ET610/60m, Chroma Tech. Co.) and detected by a CMOS camera with a 50 ms integration time.

Rab5 proteins, labeled with Alexa Fluor^™^ 633, were excited with a 640 nm laser at 251 W/cm^2^, filtered by a far-red bandpass filter (OSF-680/42, Chroma Tech. Co.), and detected accordingly. The total number of frames recorded for each cell varied between 53,000 to 118,000, depending on protein expression levels, to ensure complete imaging of all the proteins.

##### Image analysis

A custom-developed MATLAB program was employed to process fluorescence micrographs. It analyzed the fluorescence micrographs and quantified the locations of surface CTR1 and Rab5^+^ areas, including the diameters of Rab5^+^ zones. The images went through static background subtraction, PSF fitting, duplicate removal, and drift correction for accurate determination of CTR1 locations.

To remove the static background caused by gold markers or cell scattering in each image, we calculated a background image by averaging 50 images taken before and after the target image. Subtracting this from the target image yielded a background-subtracted image, which was then used to identify single-molecule events. Local bright pixels were detected using a local statistical maximum search algorithm. The local bright pixels were identified through the local maximum search algorithm. Once identified, each subregion (9 × 9 pixels) containing the fluorescent PSF was fitted with a two-dimensional (2D) Gaussian function, I(x,y), to determine the center location $\left(x_{0},y_{0}\right)$:

$$\text{I}(\text{x},\text{y})=b+e^{\left(\frac{1}{2}{\left(\frac{x-{\text{x}}_{0}}{\sigma_{\text{x}}}\right)}^{2}+{\left(\frac{y-y_{0}}{\sigma_{\text{y}}}\right)}^{2}\right)}$$

Note that the I(x,y) represents the spatial intensity distribution of the PSF; $x_{0}$ and $y_{0}$ specify the center position of the peak; $\sigma_{\text{x}}$ and $\sigma_{\text{y}}$ indicate the Gaussian function’s standard deviation, which controls the width of the bell shape.

The theoretical resolution, based on the Airy disc’s full width at half maximum (FWHM) for the PSF, is calculated using FWHM=0.51λ/NA, where λ is the wavelength and NA is the numerical aperture of the objective lens. The FWHM for mEos4b-labeled CTR1 and Rab5 were approximately 200 nm and 230 nm, respectively. Localization error for each PSF was quantified using the equation: ^54^

$$Error_{\text{i}}=\sqrt{\frac{{\text{σ}}_{\text{i}}^{2}}{N}+\frac{a^{2}}{12\times N}+\frac{8\text{π}{\text{σ}}_{\text{i}}^{4}b^{2}}{{\left(a\times N\right)}^{2}}}$$

where ${\text{σ}}_{\text{i}}$ is the standard deviation of the 2D Gaussian fit result, N is the total number of photons of the PSF, a is the pixel size, and b is the standard deviation of the background obtained by subtracting the fitted 2D Gaussian function from the background-subtracted image. For accurate representation of CTR1 locations by single-molecule PSFs, only PSFs with an FWHM between 117 to 600 nm, localization error between 0 to 20 nm, and SNR larger than 3.6 were considered in the analysis (Extended Data Fig. 3f).

#### Single-molecule neighbor density (smND) assay

The smND assay is a technique for determining the oligomeric states of proteins within cells. Using protein locations obtained from SMLM, smND calculates the density of neighboring protein molecules within a defined region, yielding the neighbor density (*ND*). This data is then used to generate a Probability of Neighbor Density (*PND*), which reflects the oligomeric states of the proteins. Critical factors, such as fluorophore activation efficiency and protein concentration, are incorporated into a theoretical model to ensure the experimental results align with actual cellular conditions. The weighted average subunit number () is used to quantify the average oligomeric state of a protein population (Supplementary Methods).

To validate the smND assay, it was applied to Tac-antigen fusion proteins with varying oligomeric states, including up to tetramers, demonstrating the assay’s ability to accurately quantify subpopulations of proteins when only two oligomeric states are present (Extended Data Fig. 4). However, when proteins exist in three different oligomeric states, integrating a kinetic model is necessary to robustly extract the subpopulations. By examining various protein subpopulations (Supplementary Table 2), smND has proven capable of detecting changes in oligomeric states under different conditions. While the assay may not provide absolute subpopulation values, it reliably defines the upper and lower limits of each population, making smND a valuable tool for studying protein oligomeric state changes within cells.

#### Analysis of global endocytosis

To quantify global endocytosis, COS7 cells were labeled with Alexa Fluor^™^ 555-conjugated WGA (Invitrogen W32464), following treatment with 100 μM CuCl_2_ for 15 minutes to induce endocytosis, the cells were fixed and imaged using a wide-field epi-fluorescence microscope (CKX53, Olympus). The size and number of internalized WGA-labeled objects were analyzed as indicators of global endocytosis. To assess the numbers and sizes of internalized vesicles, the WGA-labeled objects were excited with a mercury lamp (Olympus U-HGLGPS) and visualized in the red channel through filter sets (BP 530–550/DM 570/LP 575) (Olympus U-FGW), and the acquired images were analyzed using NIH ImageJ/Fiji software. The red images were processed by background subtraction using the rolling-ball algorithm with a radius of 50 pixels. The images were then auto-thresholded using the ‘Moments’ algorithm to generate binary masks. These segmented WGA images were analyzed using the ‘Particle Analysis’ tool, with a filter size set to an area of 0.12–4 μm^2^ and circularity of 0.04–1.00 to identify and quantify internalized WGA puncta. Bright-field images of the cells were used to determine the cell area.

To compare CTR1 density of Rab5^+^ to that of the outside Rab5 areas (Rab5^−^), COS7 cells were transfected with indicated CTR1^mE^, stained with Rab5, imaged and analyzed as prior described. Rab5^+^ objects with diameters smaller than 600 nm were selected as Rab5^+^ areas. Meanwhile, for each selected Rab5^+^ area, another non-overlapping Rab5^−^ area of the same size was randomly selected within the cells. This approach ensured equal numbers and total areas of Rab5^+^ and Rab5^−^ regions for comparison. CTR1 density for each Rab5 area was calculated by dividing the number of CTR1 molecules by the area of the Rab5 region.

### QUANTIFICATION AND STATISTICAL ANALYSIS

The study analyzed a total of 27 cells, 2776 Rab5^+^ and Rab5^−^ areas, and 815,194 CTR1 protein locations cumulatively from all four conditions. The molecular densities and oligomeric states of CTR1 were initially assessed within each Rab5^+^ or Rab5^−^ ROI across all cells with the similar CTR1 expression level were pooled and compared between the basal and copper-stressed conditions. Statistical evaluation was performed using the two-sample Student t-test in MATLAB, Statistics and Machine Learning Toolbox, “ttest2” function. The calculations and statistical analyses for global endocytosis and other biochemistry data were performed using Excel and GraphPad Prism 9. The statistics of all data were summarized in Supplementary Table 3.

## Supplementary Material

Supplement 1
