# Supplementary material for: Human transporter de-oligomerization regulates copper uptake into cells
Source: Res Sq. 2024 Dec 9:rs.3.rs-5456520. Preprint. [Version 1] doi: 10.21203/rs.3.rs-5456520/v1 (PMC11661305; doi:10.21203/rs.3.rs-5456520/v1)
Supplement: Supplement 1 [file NIHPPRS5456520V1-supplement-1.pdf]

Supplementary Files

This is a list of supplementary files associated with this preprint. Click to download.

- SI.docx
- ExtendedData.docx
